# Uncertainty quantification of computational fluid dynamics-based predictions for fractional flow reserve and wall shear stress of the idealized stenotic coronary

**DOI:** 10.3389/fcvm.2023.1164345

**Published:** 2023-11-27

**Authors:** Nghia Nguyen Ho, Kwan Yong Lee, Sang-Wook Lee

**Affiliations:** ^1^ School of Mechanical Engineering, University of Ulsan, Ulsan, Republic of Korea; ^2^ Cardiovascular Center and Cardiology Division, Seoul St. Mary's Hospital, The Catholic University of Korea, Seoul, Republic of Korea; ^3^ Cardiovascular Research Institute for Intractable Disease, College of Medicine, The Catholic University of Korea, Seoul, Republic of Korea

**Keywords:** uncertainty quantification, sensitivity analysis, non-intrusive polynomial chaos expansion method, computational fluid dynamics, fractional flow reserve, wall shear stress

## Abstract

**Introduction:**

In clinical practice, hemodynamics-based functional indices, such as fractional flow reserve (FFR) and wall shear stress (WSS), are useful in assessing the severity and risk of rupture of atherosclerotic lesions. Computational fluid dynamics (CFD) is widely used to predict these indices noninvasively.

**Method:**

In this study, uncertainty quantification and sensitivity analysis are performed for the computational prediction of WSS and FFR directly from 3D–0D coupled CFD simulations on idealized stenotic coronary models. Five geometric parameters (proximal, mid, and distal lengths of stenosis; reference lumen diameter; and stenosis severity) and two physiological parameters (mean aortic pressure and microcirculation resistance) are considered as uncertain input variables.

**Results:**

When employing the true values of stenosis severity and mean aortic pressure, a discernible reduction of 25% and 9.5% in the uncertainty of the computed proximal WSS, respectively. In addition, degree of stenosis, reference lumen diameter, and coronary resistance contributed the uncertainty of computed FFR, accounting for 41.2%, 31.9%, and 24.6%, respectively.

**Conclusion:**

This study demonstrated that accurate measurement of the degree of stenosis and mean aortic pressure is crucial for improving the computational prediction of WSS. In contrast, the reference lumen diameter, degree of stenosis, and coronary resistance are the most impactful parameters for FFR.

## 1. Introduction

Coronary artery disease (CAD) is the most common cause of mortality worldwide (1). Significant narrowing of the coronary lumen owing to endothelial plaque accumulation can interfere with the flow of oxygen-rich blood to the myocardium, thereby causing ischemic symptoms and myocardial infarction. Traditionally, a morphology-based index, the percentage reduction of arterial lumen diameter in coronary angiography images, has been used to define the severity of stenosis and as a decision-making tool for revascularization. However, multiple clinical trials have reported limited diagnostic accuracy and inferior correlation with prognostic outcomes. This finding emphasizes the need for a physiology-based functional index (2–4).

Pressure-wire-based fractional flow reserve (FFR) is defined as the maximal coronary flow to the myocardium in the presence of stenosis divided by the theoretical maximal blood flow in a normal coronary artery, and it is currently the reference standard for identifying hemodynamically significant stenotic lesions in coronary circulation (5–9). This was approximated as the ratio of the mean coronary pressure measured distal to the stenosis to the mean aortic pressure under hyperemic conditions. To measure FFR, an invasive pressure wire and pharmacological agents that induce maximal vasodilation of the microcirculation are required (10). In maximal vasodilation (hyperemia), the distal coronary pressure is directly proportional to the maximum vasodilated perfusion and coronary flow (7). FFR exhibits superior diagnostic performance compared with the morphology-based index (11, 12); however, the practical application rate of FFR in catheterization laboratories is only approximately 10%, possibly owing to potential risks during measurements (13). Noninvasive computational FFR methods have been developed as alternatives and showed promising potential with excluding the use of additional pressure wire and hyperemic agents (14). In this approach, FFR is computationally evaluated by solving the Navier-Stokes equations for coronary velocity and pressure fields using computational fluid dynamics (CFD) techniques based on 3D reconstructed vascular geometry derived from coronary CT angiography or x-ray angiography.

In addition, the local hemodynamic force, i.e., the wall shear stress (WSS) is widely accepted to play an important role in the development and progression of atherosclerosis (15, 16). In particular, high WSS is correlated with plaque rupture in severe stenosis (>70%); at minimal CAD, low WSS is associated with rapid plaque progression (17). Kumar et al. (18) reported that high-risk plaque rupture is more prevalent at the proximal segment of a stenotic lesion, which is presumed to be related to the increased time-varying structural strain caused by a high WSS. Furthermore, proximal WSS combined with FFR is shown to have an incremental prognostic value in the prediction of myocardial infarction compared with FFR alone.

With remarkable advancements in computer resources, computational modeling, and image processing techniques, CFD has been applied to predict patient-specific intravascular hemodynamics noninvasively. This approach requires input data, including patient-specific clinical data and the 3D lumen geometry of the coronary artery, which is typically reconstructed from computed tomography and x-ray angiography. However, owing to inevitable uncertainties associated with the inherent variability of physiological data, and the inaccuracy of *in vivo* measurements and lumen segmentation process of stenosis, the effect of these input uncertainties on the prediction accuracy of hemodynamics-based diagnostic indices must be quantified.

Although Monte Carlo simulation is a conventional and simple method for the analysis of uncertainty quantification (UQ), it is not feasible for complex problems because its convergence rate follows the principle of large numbers, i.e., its rate is approximately 1/√N, where N is the number of samples. Another technique developed in the recent decades to overcome the issue of long computing time is the polynomial chaos expansion (PCE) method (19, 20). The PCE approximates a random variable as a linear combination of polynomial functions of other random variables and is faster than the Monte Carlo method when the number of uncertain parameters is less than 20 (21). The PCE has been used to successfully solve various problems in fluid mechanics, including laminar boundary layer flow on a flat plate, supersonic flow over a convex corner, and inviscid flow around a three-dimensional wing, as illustrated in the study conducted by Hosder et al. (22).

Eck et al. (23) previously introduced the utilization of UQ and SA in the context of cardiovascular applications. They investigated on the variability of FFR using a 1D model with ten uncertain parameters: proximal length, stenosis length, distal length, proximal radius, stenosis radius, distal radius, hyperemic blood flow, arterial pressure, blood viscosity, and blood density. Concurrently, they applied both the Monte Carlo (MC) method and the Polynomial Chaos (PC) method to assess the merits and demerits of each technique. The findings revealed that among the uncertain inputs, stenotic radius, hyperemic flow, and arterial pressure played pivotal roles in contributing to the variance of FFR. Notably, the PC method demonstrated a substantial advantage over the Monte Carlo (MC) method. The PC method required a significantly lower number of samples—2,002 samples compared to the MC method’s 60,000 samples. Additionally, the distribution of FFR obtained through the PC method closely resembled that obtained via the MC method, but at a significantly lower computational cost.

Sankaran et al. (24) undertook a comprehensive investigation into the influence of uncertainties of minimum lumen diameter, lesion length, boundary conditions, and blood viscosity on blood flow and pressure in both an idealized stenosis model and a patient-specific model. They employed an adaptive stochastic method integrated with a data-driven approach to analyze the effects of these uncertainties. Their findings revealed that the minimum lumen diameter emerged as the most influential factor affecting hemodynamic simulations.

Gashi et al. (25) studied the effect of the model-order reduction approach (2D, 3D, steady, and unsteady) on the computationally predicted FFR and reported that stenosis severity is the dominant geometric parameter for all cases.

Although these studies provide valuable information regarding the influence of the uncertainty of geometrical and physiological features on computed FFR, they were based only on 1D model simulations or were limited to a systematic investigation of the interactive contribution of uncertain input parameters to variations in FFR output. Furthermore, information regarding the uncertainty of stenosis WSS predictions is lacking.

In this study, an investigation was conducted to assess the relative significance of input variables and the repercussions of their uncertainties on computational predictions of FFR and WSS. Utilizing uncertainty quantification (UQ) and sensitivity analysis (SA), the nonintrusive PCE method was employed. The analytical approach involved an unsteady 3D-0D coupled CFD analysis, employing idealized coronary artery models rather than a simplified 1D approach. Lumped parameter networks were used to model the blood flow within the coronary artery, with the assumption that the coronary resistance during hyperemia is a quarter of the coronary resistance in the normal state. To account for geometric uncertainties, an eccentric stenosis case was considered due to its prevalence in CAD. Seven prospective uncertain inputs were considered: proximal length, middle length, distal length, reference lumen diameter of the coronary artery (representing the healthy diameter), degree of stenosis, mean aortic pressure, and coronary resistance. Additionally, the convergence of the nonintrusive PCE method was addressed by comparing various statistical indices at different polynomial orders.

## 2. Materials and methods

### 2.1. Geometry modeling and uncertain input variables

**Figure 1:**
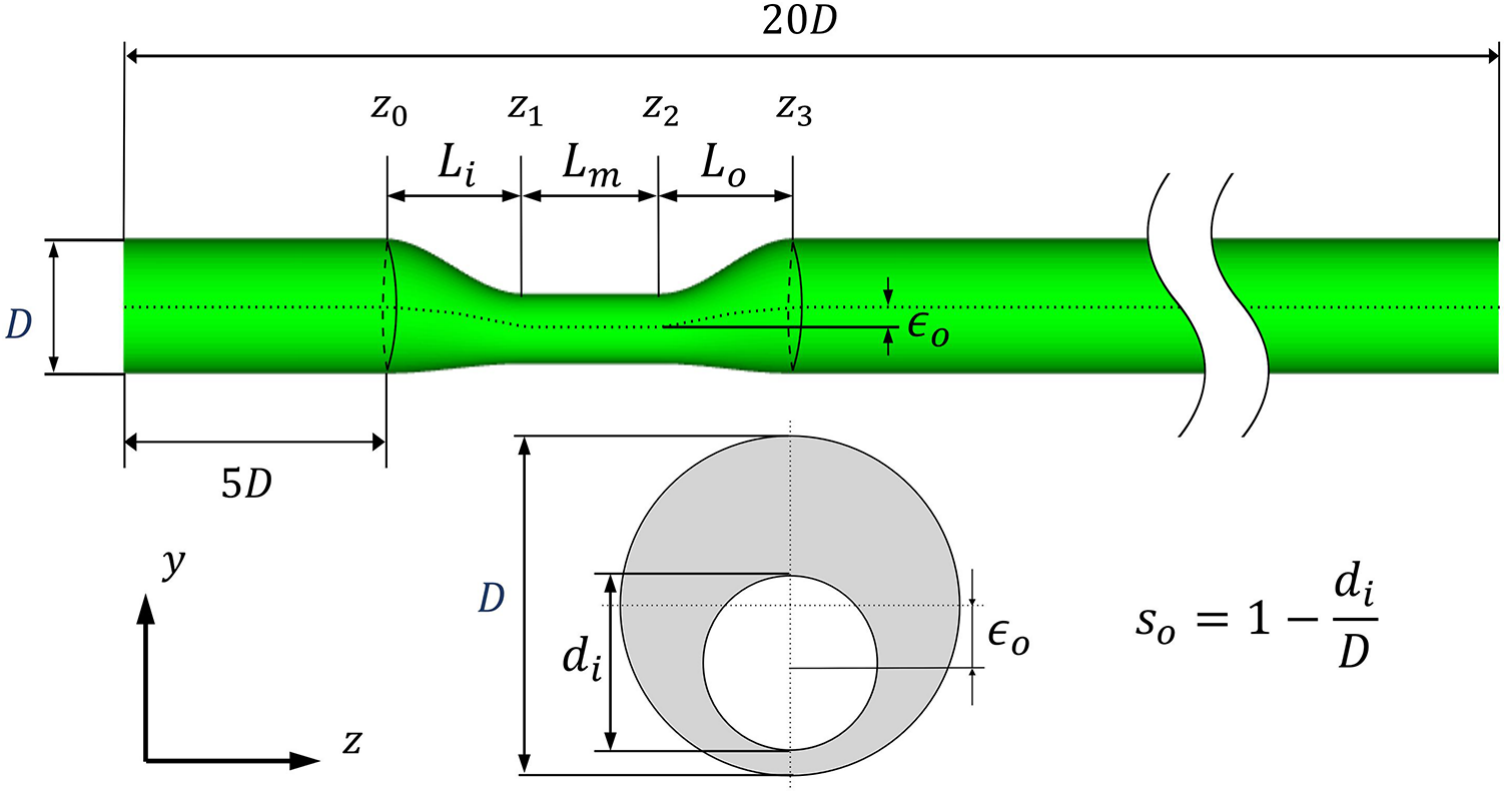
Schematic of an idealized stenotic coronary model.

To investigate the effects of uncertainty in the input variables on FFR and WSS, coronary models were generated based on a synthetically designed single conduit with various geometric parameters of stenosis, as shown in Figure 1. Five uncertain geometric features were considered: proximal, mid, and distal lengths of stenosis; reference lumen diameter of the coronary artery; and diameter-based stenosis severity. The eccentricity of the stenosis was fixed at 35% in all the models. In addition, the mean aortic pressure and normalized microcirculation resistance were included as uncertain physiological variables because the flow rate and cardiac pressure generally play important roles in coronary flow dynamics. The details of the mean value and uncertainty levels for each input variable considered in this study are presented in Table 1. The degree of uncertainty for the input variables was assumed based on the previous study, in which *in vivo* data typically observed in patients were considered (23). Mean values of vessel geometric parameters were determined by analyzing approximately 400 patients’ left anterior descending (LAD) coronary profiles from the literature (26–29). The mean aortic pressure (*P_a_*) of 90 mm Hg was applied. The microcirculation resistance (*R*) was set to match the mean flowrate of 1 ml/s under baseline conditions and was reduced by four times (*R/4*) for the hyperemic condition (30).

**Table 1:** Descriptions of uncertain input variables of the computational model.

Variable	Description	Mean	Unit	Uncertainty
*L_i_*	Proximal length of stenosis	6.0	mm	5%
*L_m_*	Middle length of stenosis	6.0	mm	5%
*L_o_*	Distal length of stenosis	6.0	mm	5%
*D*	Reference lumen diameter of the coronary artery	3.6	mm	5%
*s*_0_	Degree of stenosis	50	%	5%
*ɛ*_0_	Eccentricity of stenosis	35	%	–
*P*_a_	Mean aortic pressure	90	mmHg	10%
*R*	Normalized value of coronary resistance	1	–	10%

An idealized stenotic coronary model with a 3.6 mm-diameter was constructed based on the following equations for the geometric contours.


(1) $$d(z)=\{\;\begin{array}{c} D\left[1-s_{0}\sin\frac{\pi }{2L_{i}}(z-z_{0})\right]\;,\;\;\;z_{0}\leq z\leq z_{1}\\ D\;[1-s_{0}],\;\;\;\;\;\;\;\;\;\;\;\;\;\;\;\;\;\;\;\;\;\;\;\;\;\;\;z_{1}\leq z\leq z_{2}\\ D\left[1-s_{0}\cos\frac{\pi }{2L_{0}}(z-z_{2})\right],\;\;\;z_{2}\leq z\leq z_{3} \end{array},$$

(2) $$e(z)=\{\;\begin{array}{c} e_{0}\sin\frac{\pi }{2L_{i}}(z-z_{0})\;,\;\;\;z_{0}\leq z\leq z_{1}\\ e_{0}\left(=\epsilon_{0}s_{0}\frac{D}{2}\right)\;\;\;\;\;\;\;\;\;\;\;\;\;\;z_{1}\leq z\leq z_{2}\\ e_{0}\cos\frac{\pi }{2L_{o}}(z-z_{2}),\;\;\;z_{2}\leq z\leq z_{3} \end{array},$$

(3) $$\left[\begin{array}{c} x(z)\\ y(z) \end{array}\right]=\left[\begin{array}{c} 0\\ e(z) \end{array}\right]+\frac{d(z)}{2}\left[\begin{array}{c} \cos\theta \\ \sin\theta \end{array}\right]\;\;\;\;\;\;0\leq \theta \leq 2\pi ,$$

Here, *d(z)* and *e(z)* represent the vessel diameter at the stenotic segment and the central axis of stenosis along its length, respectively. *e*_0_ represents the maximum off-center distance of the stenotic segment; *D* is the reference lumen diameter of the coronary artery; *s*_0_ is the degree of stenosis.

The eccentricity of stenosis *ε*_0_ is defined as the percentage measure of asymmetry or off-center narrowing within the arterial lumen. Previous studies have demonstrated a higher prevalence of eccentric stenosis compared to concentric stenosis. Mintz et al. (31) reported an occurrence of 795 out of 1,446 (55%) eccentric lesions based on angiography data. Similarly, Yamagishi et al. (32) investigated the morphological characteristics of 114 coronary plaques using intravascular ultrasound and observed that 92 (80%) cases of stenosis exhibited an eccentric pattern. However, Seo et al. (33) reported that lesion eccentricity has no statistically significant effect on FFR. Based on these characteristics, the coronary model in this study considered a fixed eccentricity of 35%, which significantly reduces the CFD computation time.

Eventually, the uncertainties in the variables are propagated to the CFD model via the Latin hypercube sampling method (33) with a uniform distribution.

### 2.2. Computational fluid dynamics

3D–0D coupled CFD simulations for stenotic coronary flow were performed (34, 35). The flow was assumed to be transient, and the blood was an incompressible Newtonian fluid with a density of 1,050 kg/m^3^ and kinematic viscosity of 3.5 × 10^−6^ m^2^/s. The unsteady Navier–Stokes equations describing the conservation of mass and momentum of the blood flow are as follows.

(4) $$\rho {\dot{u}}_{i}+\rho u_{j}u_{i,j}=-p_{,i}+(\tau_{i,j}{)}_{,{\;j}^{\prime }}$$

(5) $$u_{i,i}=0.$$

For the numerical calculations, these governing equations were split into four steps for time advancement based on a fully implicit fractional step method (36) as follows.

(6) $$\;\mathrm{Step}\;1:\;\rho \frac{{\hat{u}}_{i}-u_{i}^{n}}{\Delta t}+\rho \;\frac{1}{2}({\hat{u}}_{j}{\hat{u}}_{i,j}+u_{j}^{n}u_{i,j}^{n})=-\;p_{,i}^{n}+\frac{1}{2}({\hat{\tau }}_{ij}+\tau_{ij}^{n}{)}_{,j}$$

(7) $$\;\mathrm{Step}\;2:\;\rho \frac{u_{i}^{*}-{\hat{u}}_{i}}{\Delta t}=p_{,i}^{n},$$

(8) $$\;\mathrm{Step}\;3:\;p_{,ii}^{n+1}=\frac{\rho }{\Delta t}u_{i,i}^{*},$$

(9) $$\;\mathrm{Step}\;4:\;\rho \frac{u_{i}^{n+1}-u_{i}^{*}}{\Delta t}=-\;p_{,i}^{n+1},$$

where *Δt* is the time increment; the superscript *n* indicates the time level; and ${\hat{u}}_{i}$ and $u_{i}^{*}$ are intermediate velocities. The second-order implicit Crank–Nicolson scheme was employed for the diffusion and convection terms.

**Figure 2:**
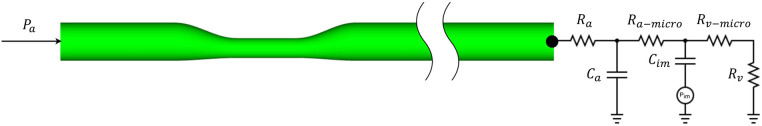
Schematic of a 3D–0D coupled CFD approach for an idealized stenotic coronary flow simulation.

To accommodate the specific time-varying pressure-flow characteristics of coronary flow, the 0D lumped parameter network (LPN) modeling technique for coronary microcirculation was integrated into 3D CFD at the outlet, as shown in Figure 2. The LPN model represents the coronary circulatory system by combining resistance and capacitance elements based on the similarity between the hydraulic and electric systems. The total resistance and capacitance of the distal coronary bed at rest (baseline) and under the hyperemic condition, which is the maximum flow condition achieved by minimizing microvascular resistance, were determined based on previous studies conducted by Sankaran (24) and Sharma et al. (37). The MPI parallel algorithm was applied to reduce the computation time to a simulation time within 1 h (about 20 min with 64 parallel cores) (35).

**Figure 3:**
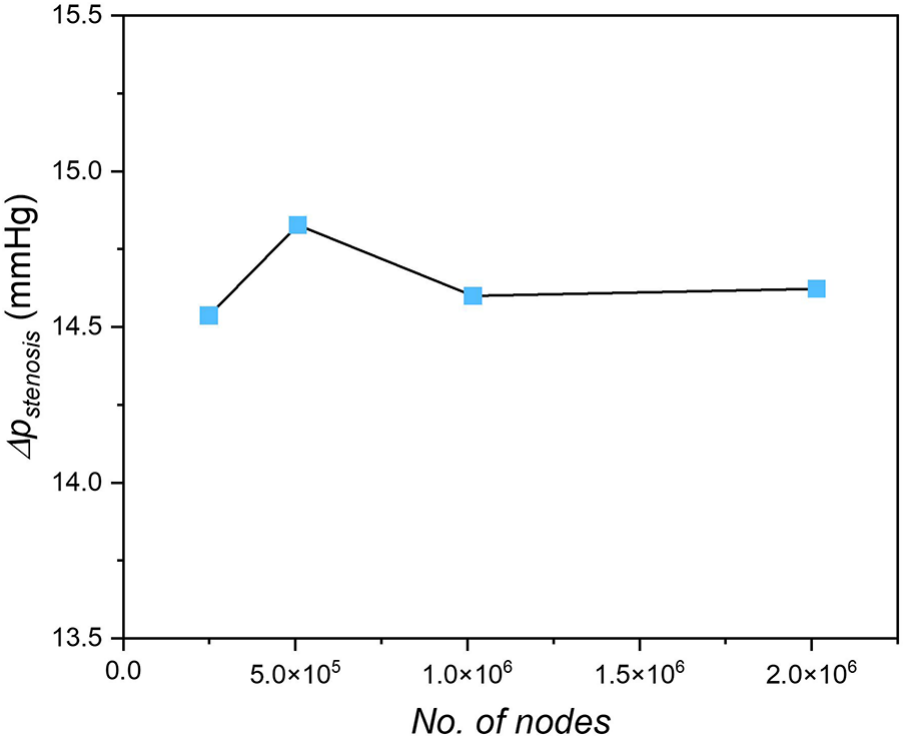
Mesh independent test for the idealized stenotic coronary.

A P2P1 finite element scheme (35) was employed with quadratic tetrahedral elements generated using a commercial mesh generator (ICEM-CFD, ANSYS) to achieve higher accuracy. A mesh convergence study was performed by comparing the stenotic pressure drop $\Delta p_{stenosis}$ in the case of different mesh densities, shown in Figure 3. The difference of $\Delta p_{stenosis}$ between the medium mesh (the number of nodes ∼1 million) and the densest mesh (the number of nodes ∼1.8 million) is within 0.5%; Meanwhile, the computation time significantly increases from 1,400 s to 3,000 s with the same computational resources (56 parallel cores, Intel Xeon E5-2630 v4). In this way, the mesh with the number of nodes of 1 million was chosen for this study.

**Figure 4:**
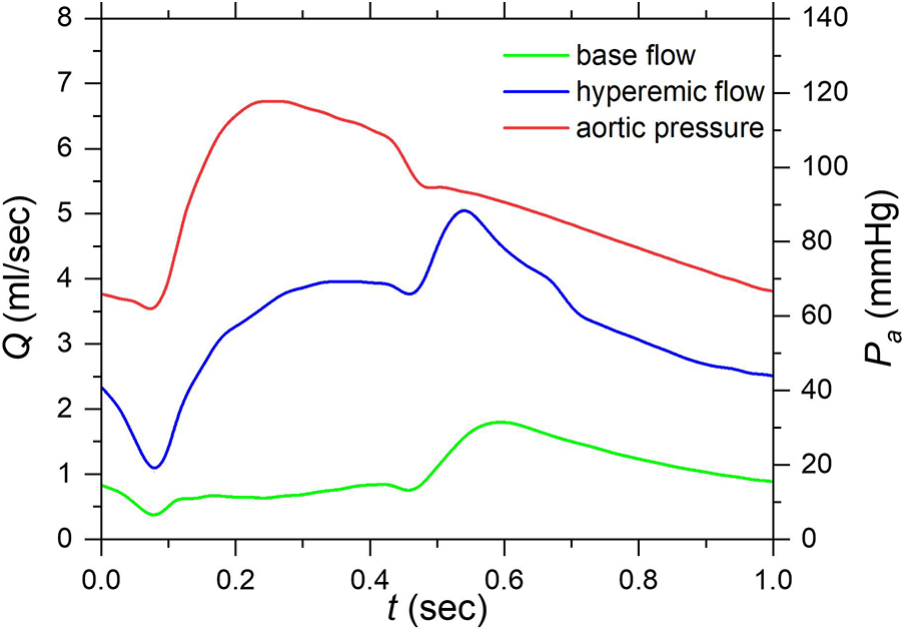
Aortic pressure and flow rate waveforms under resting and hyperemic conditions of a cardiac cycle. (Aortic pressure was applied at the inlet as a boundary condition and resting and hyperemic flow rates are computed results under corresponding conditions).

For the inlet boundary conditions, a typical aortic pressure waveform (39), scaled using a mean aortic pressure of 90 mmHg, was applied. The duration of each cardiac cycle is 1.0 s and is divided into 120 timesteps. The convergence criteria are $\;1\times 10^{-6},\;5\times 10^{-6}$ for velocity and mass, respectively. Figure 4 illustrates the blood flow rate under resting (baseline) and hyperemic conditions and the pressure waveforms for a cardiac cycle.

### 2.3. Point-collocation nonintrusive polynomial chaos method

In the nonintrusive polynomial chaos (NIPC) method, a surrogate model *f_PC_* based on polynomials for output *Y* is generated, as follows.

(10) $$Y\approx f_{\mathrm{PC}}(X)=\overset{N_{P}}{\underset{i=1}{\sum }}\alpha_{i}\Psi_{i}(X),$$

where Ψ*_i_* is the expansion or polynomial (typically orthogonal polynomial) function in relation to the probability distribution of inputs $X;\;\alpha_{i}$ is a coefficient to be computed; and *N_p_* is the total number of discretization terms.

In practice, *N_p_* is expressed by the following equation in terms of the number of uncertain input parameters *n* and the order of the polynomial *p*:

(11) $$N_{p}=\;\frac{(n+p)!}{n!p!}$$

The inputs $X((X_{i}),\;i=1,2,..,n)$ are treated as uncertain variables and hypothesized independent; therefore, the random space of uncertain inputs ***X*** is drawn by the joint probability distribution *ρ_x_*, which is consisted of other random variables *X_i_*:

(12) $$\rho_{X}(x)=\overset{n}{\underset{i=1}{\prod }}\rho_{X_{i}}(x_{i})$$

The point collocation NIPC method determines the components *a_i_* by choosing *N_s_* vectors $\left\{X_{j}={\left(X_{1},X_{2},\ldots ,X_{n}\right)}_{j},\;j=1,\;2..,N_{s}\right\}$ from the random space and subsequently evaluating the outputs of interest $\left\{Y_{j},\;\;j=1,\;2..,N_{s}\right\}$ by deterministic CFD code. Corresponding to each chosen vector there will be an output, a linear system of equations can be established:

(13) $$\left(\begin{array}{cccc} \Psi_{1}(X_{1}) & \Psi_{2}(X_{1}) & \ldots & \Psi_{N_{P}}(X_{1})\\ \Psi_{1}(X_{2}) & \Psi_{2}(X_{2}) & \ldots & \Psi_{N_{P}}(X_{2})\\ \vdots & \vdots & \ddots & \vdots \\ \Psi_{1}(X_{N_{s}}) & \Psi_{2}(X_{N_{s}}) & \ldots & \Psi_{N_{P}}(X_{N_{s}}) \end{array}\right)\left(\begin{array}{c} \alpha_{1}\\ \alpha_{2}\\ \vdots \\ \alpha_{N_{P}} \end{array}\right)=\;\left(\begin{array}{c} Y_{1}\\ Y_{2}\\ \vdots \\ Y_{N_{s}} \end{array}\right)$$

(14) $$\alpha =\mathrm{argmi}n_{\alpha }\|\Psi \alpha -Y{\|}_{2}$$

The number of chosen samples *N_s_* should be equal or greater than number of discretization terms *N_p_*; in the case of $N_{s}>N_{p}$, the deterministic problem becomes to over-determined system of equations (Eq. 13), then the least squares method can be used to solve (Eq. 14). Hosder et al. (22) suggested that twice as many samples as the minimum (practical) are required to better approximate the statistics at each polynomial degree $\left(N_{s}=2N_{p}\right)$. Here, $n_{\;ps}$ is denoted as the proportion of sample:

(15) $$n_{\;ps}=\;\frac{N_{s}}{N_{p}}$$

To apply the NIPC method for FFR and WSS, the uncertain input parameters are predefined with a probability distribution, then these parameters are sampled and applied to a deterministic solver to obtain outputs of interest (FFR and WSS); finally, the calculation of UQ and SA was performed. In this study, uncertain input parameters were identified as geometric $\left(L_{i},\;L_{m},L_{o},D,s_{0}\right)$ and physiological $\left(P_{a},\;R\right)$ features. The uncertain parameters were hypothesized independently distributed and sampled with joint distribution by Latin hypercube to select the collocation points; well-validated in-house CFD code was used to calculate FFR and WSS (35).

The outputs of interest (here, FFR and WSS) are considered as random variables. Practically, the probability density functions of FFR and WSS $\left(\rho_{FFR},\mathrm{and}\;\rho_{WSS}\right)$ are dependent on the uncertain inputs $\left(L_{i},\;L_{m},L_{o},D,s_{0},P_{a},\;R\right)$, which are governed by the CFD model. The characteristics of $\rho_{FFR}$ and $\rho_{WSS}$ were assessed using uncertainty quantification (UQ) technique, and the contribution of an uncertain input $X_{i}$ to the outputs of interest was evaluated using the sensitivity analysis (SA).

The convergence rate generally depends on both the number of samples *N_s_* and the order of the polynomial *p*. Four different maximal polynomial orders were considered (p=1,2,3and4), with the proportion of sample $n_{\;ps}=1\;\mathrm{and}\;2$ were used to assess the convergence of UQ measures.

The Python package Chaospy (38) was used to generate stochastic samples and calculate the surrogate model for the simulation output of interest.

### 2.4. Uncertainty quantification and sensitivity analysis

Characteristics of unknown distribution of *Y* using UQ are described as statistical moments (e.g., mean and variance). In the NIPC method, a surrogate model *f_PC_* consisting of orthogonal polynomials used to replace *Y*; therefore, the mean of unknown distribution of *Y* can be directly obtained using the coefficients and the orthogonality property. For example, the mean and variance of *Y* are given by:

(16) $$\mu [Y]=E[Y]=\underset{\Omega }{\int }y\rho (y)dy=\underset{\Omega }{\int }\overset{N_{P}}{\underset{i=1}{\sum }}\alpha_{i}\Psi_{i}(x)\rho_{X}(x)dx=\alpha_{1}$$

(17) $$\begin{array}{c} Var(Y)=E[Y^{2}]-E{\left[Y\right]}^{2}=\;\underset{\Omega }{\int }{\left(\overset{N_{P}}{\underset{i=1}{\sum }}\alpha_{i}\Psi_{i}(x)\right)}^{2}\rho_{X}(x)dx-\alpha_{1}^{2}\\ =\overset{N_{P}}{\underset{i=1}{\sum }}\alpha_{i}^{2}\underset{\Omega }{\int }\Psi_{i}{\left(x\right)}^{2}\rho_{X}(x)dx-\alpha_{1}^{2}=\overset{N_{P}}{\underset{i=2}{\sum }}\alpha_{i}^{2}\underset{\Omega }{\int }\Psi_{i}{\left(x\right)}^{2}\rho_{X}(x)dx \end{array}$$

The contribution of the uncertain input parameters to the variation in the output was investigated by sensitivity analysis. The final goal is determining how the uncertainties in the input variables contribute to the variance of the output, either individually or through interactions with other parameters. This is useful for modeling personalization to determine the parameters that need to be optimized to their true values (input prioritization) and those that can be fixed within their uncertainty domains (input fixing). The main and total sensitivity indices ($S_{i}$ and $S_{i}^{T}$, respectively) (23) were computed to represent input prioritization and input fixing, respectively.

The main sensitivity index, also called the first-order Sobol sensitivity index, is the variance of the conditional expectation of output *Y*, given the value of input $X_{i}$, normalized by the total variance:

(18) $$S_{i}=\;\frac{\mathrm{Var}[E[Y|X_{i}]]}{\mathrm{Var}[Y]}.$$

Here, the index *i* varies from 1 to the number of uncertain input variables n,1≤i≤n.

The main sensitivity index $S_{i}$ represents the expected reduction in the total variance Var[Y] when the input variable $X_{i}$ is corrected to its true value.

The total sensitivity index $S_{i}^{T}$ includes the sensitivity of both the first-order effects and the interactions (covariance) between a given parameter $X_{i}$ and all other parameters:

(19) $$S_{i}^{T}=\;1-\frac{\mathrm{Var}[E[Y|X_{-i}]]}{\mathrm{Var}[Y]},$$

where $X_{-i}$ is the set of all uncertain input variables except $X_{i}$.

The convergence of the UQ measures was assessed by evaluating the mean value obtained with different polynomial orders *p* and the proportion of sample $n_{\;ps}$.

## 3. Results

**Figure 5:**
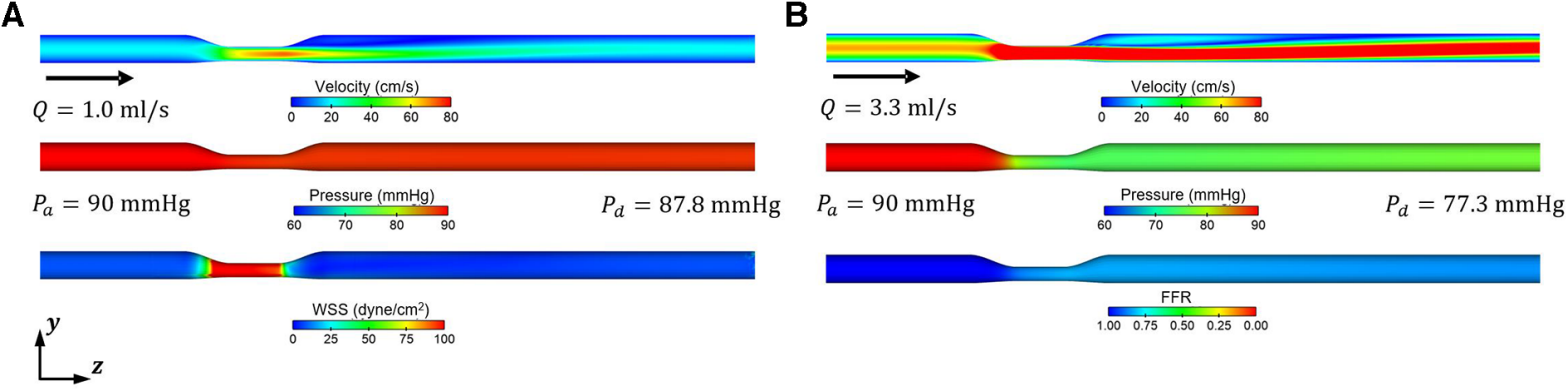
Representative blood flow and pressure fields obtained from CFD simulations for resting (baseline) and hyperemic conditions. (**A**) Baseline condition (*R_base_*) (**B**) Hyperemic condition $\left(R_{hyper}=\;0.25\;\times R_{base}\right)$.

The blood flow and pressure fields for the mean values of the input variables in the baseline and hyperemic conditions from the CFD simulations are shown in Figure 5. An increase of approximately 3.3-fold in the mean flow rate was observed when the microvascular resistance was decreased by a factor of four relative to the baseline condition to model the hyperemic condition. In addition, a significant difference of 2.2 mmHg and 12.7 mmHg was observed in pressure drop between baseline and hyperemic conditions. This difference is primarily due to increased flow separation at higher flowrates and possibly turbulence.

### 3.1. UQ and SA for WSS

Slager et al. (39) reported a tendency to form high-risk plaques localized in stenotic proximal segments with a high WSS. Herein, UQ and SA were conducted to investigate the impact of uncertain input variables on the uncertainty of the average proximal WSS $\left(AWSS_{\;prox}\right)$. A surrogate model was derived from 3D CFD data using PCE.

**Figure 6:**
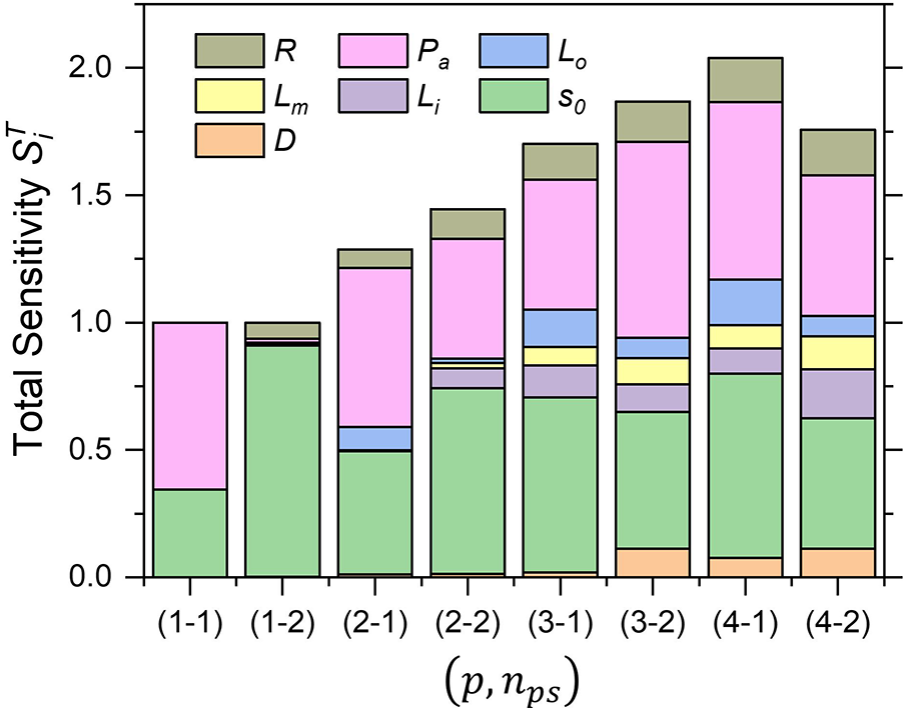
Trend of total sensitivity indices of *AWSS_prox_* for each combination of polynomial order and sample size (*p*, *n_ps_*).

Figure 6 shows a comparison of the total sensitivity index $S_{i}^{T}$ of $AWSS_{\;prox}$ for various combinations of polynomial order *p* and sample size $n_{\;ps}$. The behavior of $S_{i}^{T}$ exhibited a similar trend for $\left(\;p,n_{\;ps}\right)$ combinations higher than $\left(\;p=3,n_{\;ps}=2\right)$, which suggests suitable convergence.

**Figure 7:**
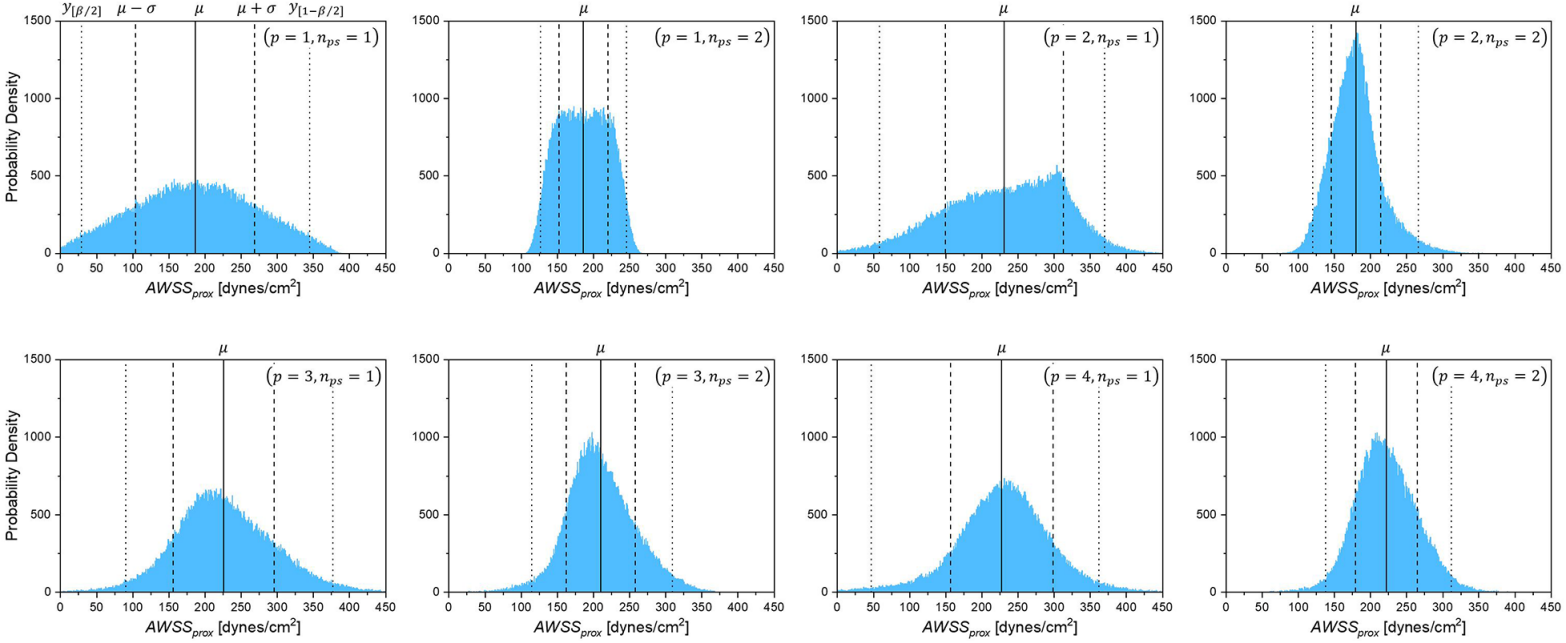
Probability density of *AWSS_prox_* for each combination of polynomial order and sample size (*p*, *n_ps_*).

The degree of stenosis *S_o_* and the mean aortic pressure $P_{a}$ were always in the highest-sensitivity group, with a main sensitivity greater than 50%, whereas the other variables ranged from 10% to 20%. In addition, the probability density distributions for each combination of $\left(\;p,n_{\;ps}\right)$ were compared, as shown in Figure 7. The probability density distribution by $\left(\;p=3,n_{\;ps}=2\right)$ combination exhibits nearly the same asymmetric shape as the one by $\left(\;p=4,n_{\;ps}=2\right)$ combination with only minor difference of the mean and standard deviation, which indicates the results with the number of polynomials p=3 and p=4 approach the convergence, particularly, when oversampled as $n_{\;ps}=2$. However, it is clearly exhibited that the proportion of sample, $n_{\;ps}=1$ is not sufficient for the approximation of the statistics, even though high polynomial order p=4 is applied.

**Figure 8:**
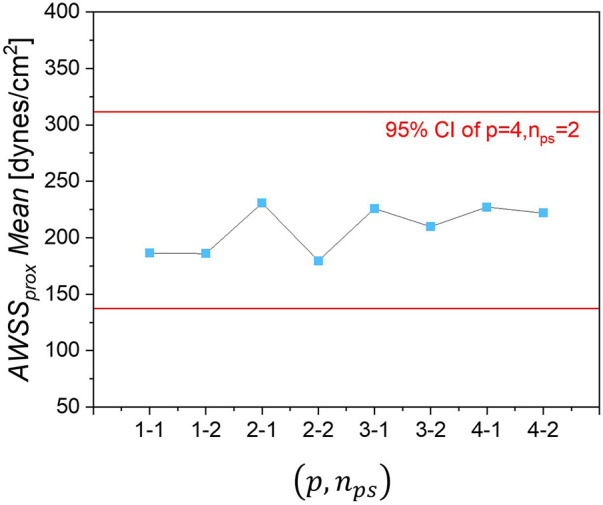
Mean of *AWSS_prox_* for each combination of polynomial order and sample size (*p*, *n_ps_*).

The mean of $AWSS_{\;prox}$ obtained with different combinations of $\left(\;p,n_{\;ps}\right)$ are given in Figure 8, showing that the obtained mean value tends to converge (relative error < 7.8%) as the polynomial order increases.

Table 2 lists the required number of samples (3D CFD realizations) and the corresponding computing times associated with each combination $\left(\;p,n_{\;ps}\right)$. Based on this observation, the combination $\left(\;p=3,n_{\;ps}=2\right)$ is optimal in terms of the computational cost and prediction accuracy.

**Table 2:** Number of samples (computing time; hours) for each combination of (*p*, *n_ps_*).

*p* *n_ps_*	1	2	3	4
1	8 (2.7)	36 (12)	120 (40)	330 (110)
2	16 (5.3)	72 (24)	240 (80)	660 (220)

**Figure 9:**
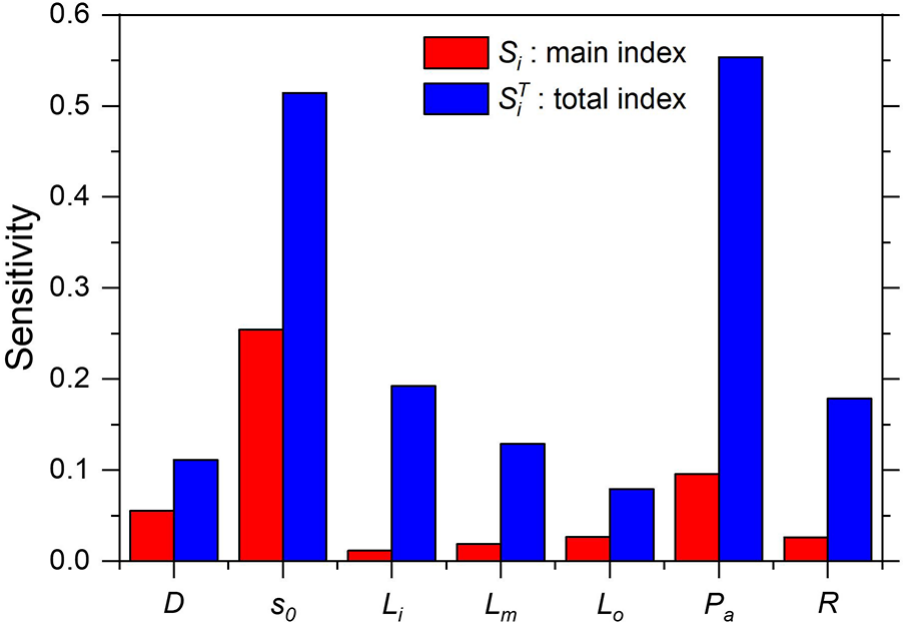
Sensitivity indices for computed *AWSS_prox_*.

As shown in Figure 9, the stenosis severity $s_{0}$ exhibited the highest main sensitivity index, followed by the mean aortic pressure $P_{a}$, whereas the remaining variables exhibited only a minor effect (≤5%). More precisely, these sensitivity indices indicate that if *s_o_* and $P_{a}$ are changed to their true values, the uncertainty in $AWSS_{\;prox}$ prediction is reduced by 25% and 9.5%, respectively. The total sensitivity index exhibited a trend similar to that of the main sensitivity index, that is, the greatest influence was from stenosis severity $s_{0}$ and mean aortic pressure $P_{a}$. However, the difference between the main and total sensitivity indices was considerably large. This indicates that the interaction of the input variables in the computational model for WSS prediction is significant; thus, multivariate uncertainty analysis is crucial.

### 3.2. UQ and SA for FFR

For FFR computations, the same conditions for uncertain input variables were applied, except for microvascular resistance, which was decreased by a factor of 4 relative to the baseline condition, to ensure a hyperemic condition. The FFR was evaluated 20 mm downstream of the stenosis in the pressure fields from the CFD simulations.

**Figure 10:**
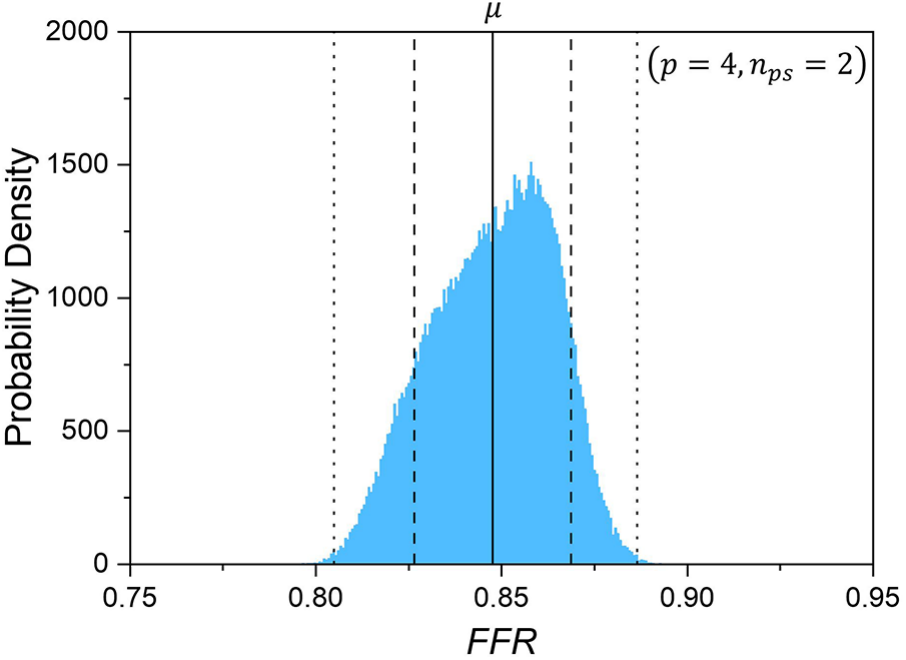
Probability density of computed *FFR*.

Figure 10 shows the statistical characteristics of the probability density of the FFR due to the uncertainty of the input parameters, including the expected values, standard deviation, and 95% prediction interval. Evidently, the distribution is asymmetric and left-skewed toward lower FFR values, unlike $AWSS_{\;prox}$ which is right-skewed toward higher FFR values.

**Figure 11:**
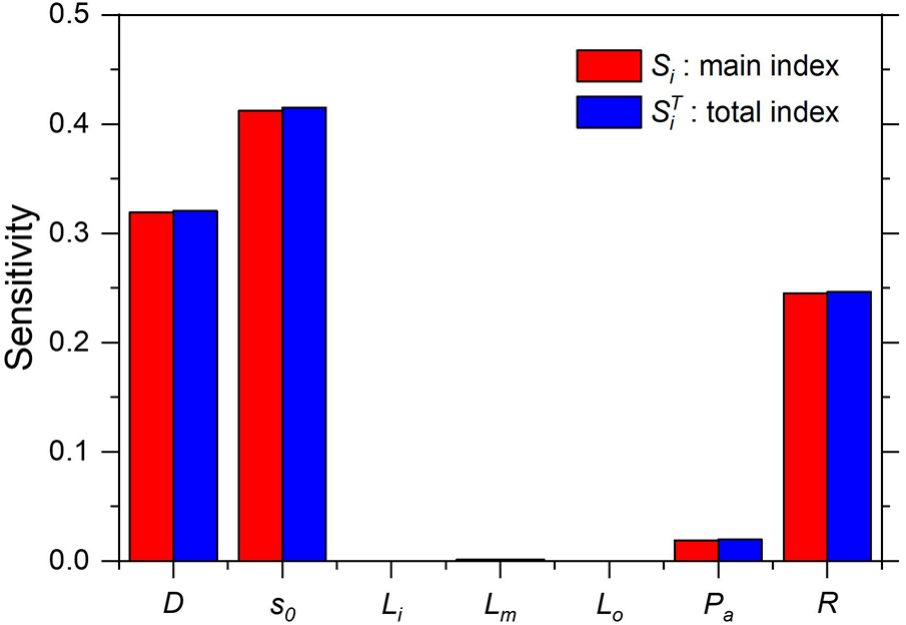
Sensitivity indices for computed *FFR*.

Figure 11 shows that only the coronary reference diameter *D*, stenosis severity $s_{0}$, and microvascular resistance *R* significantly affected the uncertainty in the FFR prediction. However, the influence of the mean aortic pressure was <0.05. The difference between the main and total sensitivity indices was nearly zero, thus indicating a negligible interaction of the input variables that are independent in the computational model for FFR prediction.

## 4. Discussion

Herein, UQ and SA were performed for the computational prediction of the WSS and FFR directly from 3D–0D coupled CFD simulations of idealized stenotic coronary models. The mean degree of stenosis was 50% based on diameter reduction. Five geometric parameters for the coronary stenosis model (proximal, mid, and distal lengths of stenosis; reference lumen diameter; and diameter-based stenosis severity) and two physiological parameters (mean aortic pressure and normalized microcirculation resistance) were considered as uncertain input variables for the CFD simulations. Although viscosity and density may be additional blood characteristics that influence the WSS and FFR values, they were not included in this study because the uncertainty of the parameters is not significant. Furthermore, Sankanran et al. (24) reported that viscosity and density have negligible effects on the uncertainty of the computed FFR.

Eck et al. (23) demonstrated that the stenotic radius, hyperemic flow rate, and arterial pressure contributed the most to the uncertainty in the FFR using 1D model-based simulation data. This finding is consistent with the results of the present study. Herein, the reference vessel diameter, stenosis severity, and microvascular resistance were found to exhibit the greatest impact on FFR prediction. Microvascular resistance under hyperemic conditions primarily determines the coronary hyperemic flow rate. In clinical practice, the FFR value is measured under the assumption of zero microvascular resistance after inducing a hyperemic condition with a drug; in reality, the value is decreased by three–five times owing to the resistance, which varies among patients. In the present study, arterial pressure influenced the uncertainty of FFR prediction; however, the relative sensitivity was low.

Sankaran et al. (24) showed that the minimum lumen diameter and outlet boundary resistance are the most important factors contributing to the variance of the computed FFR, and that their influence is independent and not interactive. Evidently, similar results were observed herein because the combined reference diameter and degree of stenosis were directly associated with the minimum lumen diameter. The interactive effect of input uncertainty on the FFR was also negligible.

For the average proximal WSS $\left(AWSS_{\;prox}\right)$, the mean aortic pressure had the greatest impact on the prediction uncertainty. This is because the WSS was calculated in the baseline flow condition, and we expect that aortic pressure has a greater effect on the baseline flow rate with higher outlet resistance than in a hyperemic condition. In addition, because WSS is inversely proportional to the third power of the lumen radius, stenosis severity has a large impact on the uncertainty of $AWSS_{\;prox}$.

Although the uncertainty of segmental lengths of a stenosis, such as $L_{i}$, $L_{m}$, and $L_{o}$, appears to have a negligible influence on the uncertainty of the computed FFR (indicating that precise measurements of the stenosis length in the imaging and reconstruction processes are not crucial), they exhibited relatively higher sensitivity to the variation of the computed $AWSS_{\;prox}$. In particular, the total sensitivity index of the proximal length of stenosis $L_{i}$ was approximately the same as that of microvascular resistance in the computed $AWSS_{\;prox}$. This implies strong interactions between the length of the stenosis and other input parameters, which should not be excluded from the distinctive input parameters in the computational prediction of WSS.

**Figure 12:**
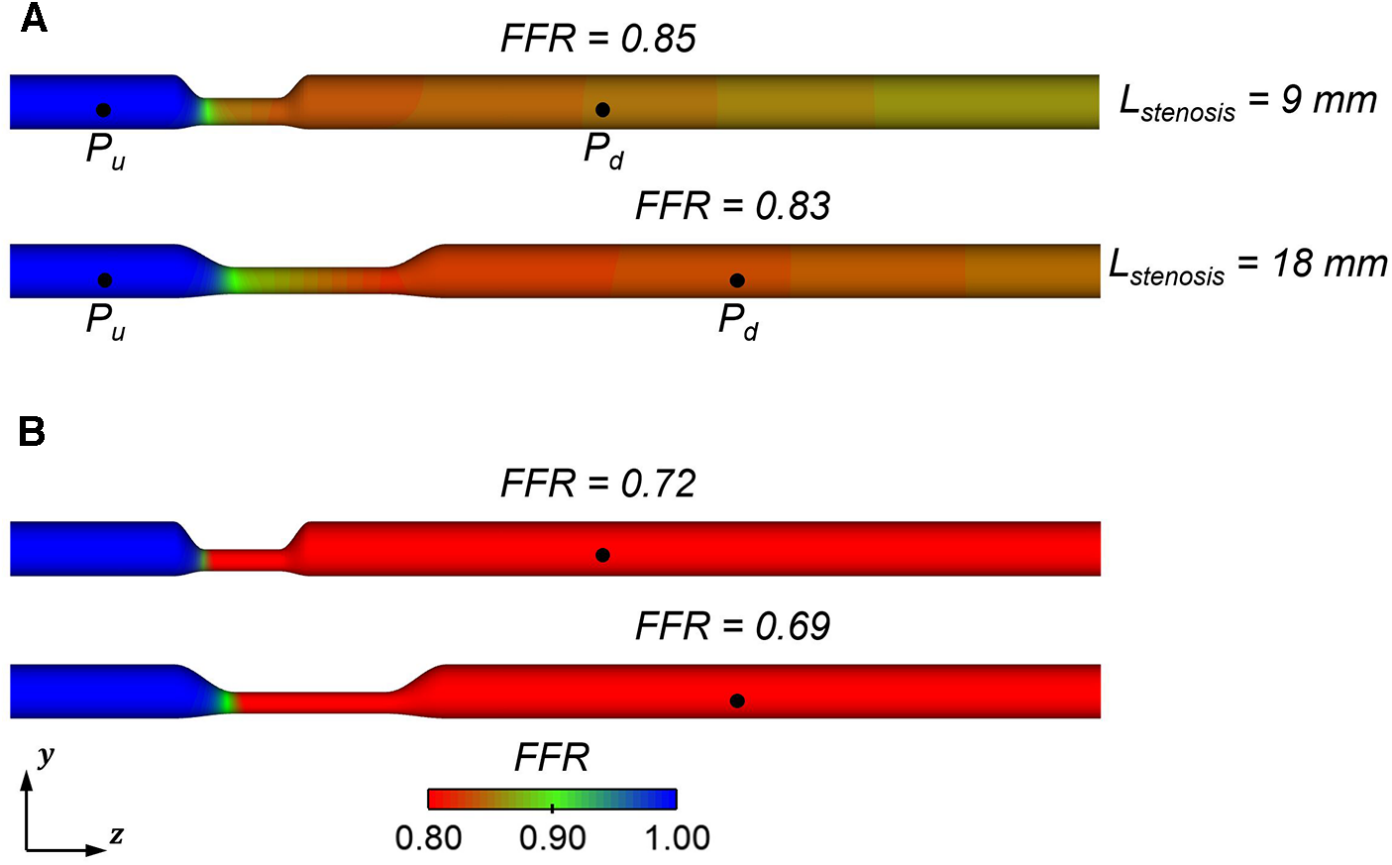
Comparison of computed FFR with different stenosis lengths (9 mm and 18 mm) (**A**) $s_{0}=50\;\text{\%}$ (**B**) $s_{0}=60\;\text{\%}$.

Moreover, the wide inter-lesion variations in patients may produce a discernible difference in FFR. As depicted in Figure 12 as a representative case, doubling the stenosis length of a stenotic vessel while preserving the other input parameters produced a notably large pressure drop and in turn a lower FFR. This suggests that the stenosis length, with appreciation of the presence of wide interlesion variations, plays a crucial role in the determination of FFR and can be considered an independent geometric factor.

## 5. Conclusion

In this study, UQ and SA were performed to quantify the effect of uncertainty of the geometric and physiological input parameters including proximal, mid, and distal lengths of stenosis, reference lumen diameter, stenosis severity, mean aortic pressure and microcirculation resistance, on the computational prediction of the WSS and FFR with an idealized stenotic coronary model. The degree of stenosis and the mean aortic pressure exhibited highest sensitivity to variations in the computed WSS. When employing the true values of stenosis severity and mean aortic pressure, a discernible reduction of 25% and 9.5% in the uncertainty of the computed proximal WSS, respectively. In addition, degree of stenosis, reference lumen diameter, and coronary resistance are the primary contributors to the uncertainty of computed FFR, accounting for 41.2%, 31.9%, and 24.6%, respectively. In particular, the interactive effect of the input variables on the uncertainty of the computed WSS is significantly higher than that on the uncertainty of the computed FFR.

## Data Availability

The raw data supporting the conclusions of this article will be made available by the authors, without undue reservation.
